# Emergence and Autochthonous Transmission of Dengue Virus Type I in a Low-Epidemic Region in Southeast China

**DOI:** 10.3389/fcimb.2021.638785

**Published:** 2021-03-24

**Authors:** Yi Zhang, Hongyi Chen, Jingen Wang, Shumei Wang, Jing Wu, Yang Zhou, Xinyu Wang, Feibing Luo, Xianglin Tu, Qiubo Chen, Yanxia Huang, Weihua Ju, Xuping Peng, Jianfeng Rao, Li Wang, Ning Jiang, Jingwen Ai, Wenhong Zhang

**Affiliations:** ^1^ Department of Infectious Diseases, National Clinical Research Center for Aging and Medicine, Huashan Hospital, Fudan University, Shanghai, China; ^2^ Department of Infectious Disease, Nanchang Ninth Hospital, Nanchang, China; ^3^ Department of Infectious Disease, Zhangshu People’s Hospital, Yichun, China; ^4^ Department of Infectious Disease, Fengcheng People’s Hospital, Yichun, China; ^5^ State Key Laboratory of Genetic Engineering, School of Life Science, Fudan University, Shanghai, China; ^6^ Key Laboratory of Medical Molecular Virology (MOE/MOH) and Institutes of Biomedical Sciences, Shanghai Medical College, Fudan University, Shanghai, China

**Keywords:** dengue fever, outbreak, transmission, metagenomic sequencing, prevention

## Abstract

**Background:**

Dengue fever is a mosquito-borne febrile illness. Southeast Asia experienced severe dengue outbreaks in 2019, and over 1000 cases had been reported in Jiangxi, a previously known low-epidemic region in China. However, the emergence of a dengue virus epidemic in a non-epidemic region remains unclear.

**Methods:**

We enrolled 154 dengue fever patients from four hospitals in Jiangxi, from April 2019 to September 2019. Real-time PCR, NS1 antigen rapid test, and IgM, IgG tests were performed, and 14 samples were outsourced to be sequenced metagenomically.

**Results:**

Among the 154 cases, 42 were identified as imported and most of them returned from Cambodia. A total of 113 blood samples were obtained and 106 were identified as DENV-1, two as DENV-2, and five were negative through RT-PCR. All DENV-1 strains sequenced in this study were all classified to one cluster and owned a high similarity with a Cambodia strain isolated in 2019. The evolutionary relationships of amino acid were consistent with that of nucleotide genome result. The sequence-based findings of Jiangxi strains were consistent with epidemiological investigation.

**Conclusion:**

Epidemiological analysis demonstrated that the emergence of dengue cases led to autochthonous transmission in several cities in Jiangxi, a low-epidemic region before. This study emphasized future prevention and control of dengue fever in both epidemic and non-epidemic regions.

## Introduction

Dengue fever is a mosquito-borne febrile illness affecting more than 100 countries in tropical and subtropical areas (Bhatt et al., 2013; Guzman et al., 2016). The introduction of *Aedes* is linked to the circulation of four serotypes of dengue viruses (DENV 1-4) globally (Guzman et al., 2016). High temperatures, humid climate, mosquitoes, globalization, and domestic or international travel increase the incidence and transmission of dengue infection (Murray et al., 2013). In most patients, dengue fever is self-limiting; however, severe dengue fever cases such as dengue hemorrhagic fever and dengue shock syndrome (DHF/DSS) could pose a life-threatening danger to infected patients (Guzman et al., 2016).

Southeast Asia experienced a severe dengue outbreak in 2019. Dengue fever remains a significant public health problem in Cambodia, with more than 10000 cases annually and have affected more than 20000 cases by July 2019 (Cousien et al., 2019). The Philippines declared a ‘National epidemic’ after the death toll from dengue fever surpassed 1000 people, most of whom were children aged < 10 years (Dyer, 2019). The Philippines reported more than 249,000 dengue fever cases, almost twice as many as those reported in 2018 (WHO, 2019). Further, dengue cases continue to surge across other southeast Asian countries (Vietnam, Bangladesh, Malaysia, Myanmar, Singapore, etc.) (**Supplementary Table 1**). The situation will deteriorate with increasing travel from cities to rural areas and from southeast Asia to other countries, giving rise to serious pandemic events (Lai et al., 2018; Masika et al., 2020).

Since the 1970s, several outbreaks have occurred in southern China, including Guangdong, Fujian, Hainan, and Yunnan provinces (Huang et al., 2014; Zhang et al., 2014; Wang et al., 2015; Li et al., 2016; Sun et al., 2017). Reemergence and autochthonous transmission originating from travelers have been reported in Hubei and Zhejiang in China (Wang et al., 2015). In China, the overall rise in the dengue fever cases has been observed (**Supplementary Figure 1**). Jiangxi, a southwest province in China, is adjacent to Fujian and Zhejiang in the east and Guangdong in the south. Located in a subtropical region, Jiangxi has a humid climate and receives plenty of sunshine and rainfall. Previously, only scattered cases of imported dengue fever cases from southeast Asia have been identified; however, this province have experienced an outbreak since June 2019.

Over 1000 dengue fever cases have been reported in Jiangxi Province in 2019. However, the emergence of a dengue virus outbreak in a non-epidemic province remains unclear. To further investigate the origin of the 2019 Jiangxi dengue outbreak, we analyzed the clinical and epidemiological characteristics of dengue fever in Jiangxi using metagenomic next-generation sequencing.

## Materials and Methods

### Participant Enrollment and Specimen

We recruited participants with clinically diagnosed or laboratory-confirmed dengue virus infections from four hospitals in Jiangxi Province, China, from April 2019 to September 2019. The participating medical facilities included Nanchang Ninth Hospital, Zhangshu People’s Hospital, Fengcheng People’s Hospital, and Nanchang Xian People’s Hospital. Blood samples on the day of admission were collected from the participants who provided their informed consent, and all recruited patients or their guardians provided their authorization for the collection of clinical data. Laboratory-confirmed cases were identified if a blood sample tested positive by either the non-structural protein (NS1) rapid test, NS1-capture enzyme linked immunosorbent assay, IgM/IgG antibody test, or reverse transcription polymerase chain reaction (RT-PCR). In contrast, clinically diagnosed cases were identified based on various symptoms, including fever, muscle or joint pains, rash, bleeding, or gastrointestinal symptoms. We collected the patients’ medical records, including clinical presentations, laboratory results, treatment regimens, epidemiological history, and follow-up monitoring. To further compare the viral phylogenic analysis, two blood samples were retrieved from Huashan Hospital, Shanghai. Ethical approval was obtained from the ethical committee of Huashan Hospital.

### Etiology Tests and RT-PCR Relative Quantification

At the beginning of this outbreak, we carried out rapid NS1 antigen and antibody IgM and IgG tests in some samples to assist diagnosis apart from RT-PCR (**Supplementary Table 2**). Since the local hospitals did not perform antibody tests in Fengcheng and Zhangshu patients and no enough volume of some samples, the antibody data are missing.

All obtained blood samples were prepared for RT-PCR test. We extracted total viral RNA from 200 µL of patients’ serum using TaKaRa MiniBEST Viral RNA/DNA Extraction Kit (TaKaRa, Japan). RNA was converted to cDNA using the TaKaRa PrimeScript RT Master Mix kit (TaKaRa, Japan). We performed real-time PCR using the BioGerm Dengue kit (BioGerm, Shanghai, China) with Taqman Probe (Haddar et al., 2020), including DENV-1/2 and DENV-3/4.

### Metagenomic Next-Generation Sequencing

A total of 14 samples from Jiangxi with relatively high dengue virus copies and two samples from Huashan Hospital were prepared for high-throughput sequencing. Then, 60 μL cDNA of each sample and a blank control were purified with 60 μL magnetic beads (MGI, Shenzhen, China). Purified cDNA samples were fragmented into approximately 150 bp (Covaris M220 Focused-ultrasonicator, Massachusetts, US), followed by end-repair, A-tailing addition, adaptor-ligation, and PCR amplification (MGIEasy Cell-free DNA Library Prep Kit, MGI, Shenzhen, China). Qualified cDNA libraries were sequenced by a single-end 100 bp sequencing strategy on the MGISEQ-200 platform (MGI, Shenzhen, China).

### Alignment and SNP Detection

Low-quality and short (average base quality < 20, length < 100 bp) reads were discarded, and reads derived from human genome sequences were filtered with Tophat2 (Kim et al., 2013). For genetic polymorphism analysis, sequenced reads were aligned to the reference isolate DENV-1 genome MF033254 using Bowtie2 with no more than 2 mismatches, and then only the uniquely mapped reads were used for genotyping analysis (Langmead and Salzberg, 2012). The Samtools mpileup and bcftools algorithms were used to generate consensus sequences for each sample. Meanwhile, amino acid sequences were aligned to DENV-1 amino acid sequence NP059433 using the multiple sequence alignment algorithm ClustalX2 (Chenna et al., 2003). A total of 56 complete dengue virus type I genomes isolated from 1956 to 2019 and respective amino acid sequences were both downloaded from the NCBI database.

### Phylogenic Tree Construction and Evolutionary Analysis

The phylogenetic tree was constructed according to the detected SNPs of all 69 sequenced and published dengue nucleotide sequences. As suggested by jModelTest (v2.1.10), we estimated the substitution rate based on the Maximum-likelihood method under the GTR+γ+I substitution model (Darriba et al., 2012). The evolutionary distances were calculated based on the similarity of the SNP genotypes and validated by at least 1000 bootstrap analyses using MEGA X (Kumar et al., 2018). The evolutionary distances results were used to annotate the phylogenetic tree in iTOL v 4 (Letunic and Bork, 2019). We used the ‘*ape*’ package in RStudio v 1.2 to analyze similarities of dengue amino acid based on polymorphic sites detected in amino acid sequences. We have uploaded the metagenomic next-generation sequence data to NCBI SRA database under accession number SRP269041.

### Epidemiological Investigations

We collected epidemiological data concerning the mosquito’s density (Breteau Index) and climate characteristics from previously published studies (Wu et al., 2017; Guo et al., 2019; Zhao et al., 2020). Further, we recorded the monthly average temperature and rain value and flight information in Jiangxi.

### Statistical Analysis

For baseline characteristics and clinical presentations, numbers and percentages were used. Figures were constructed using the RStudio v 1.2 and GraphPad Prism 8.

## Results

### Clinical and Epidemiologic Characteristics of Enrolled Patients

**Figure 1:**
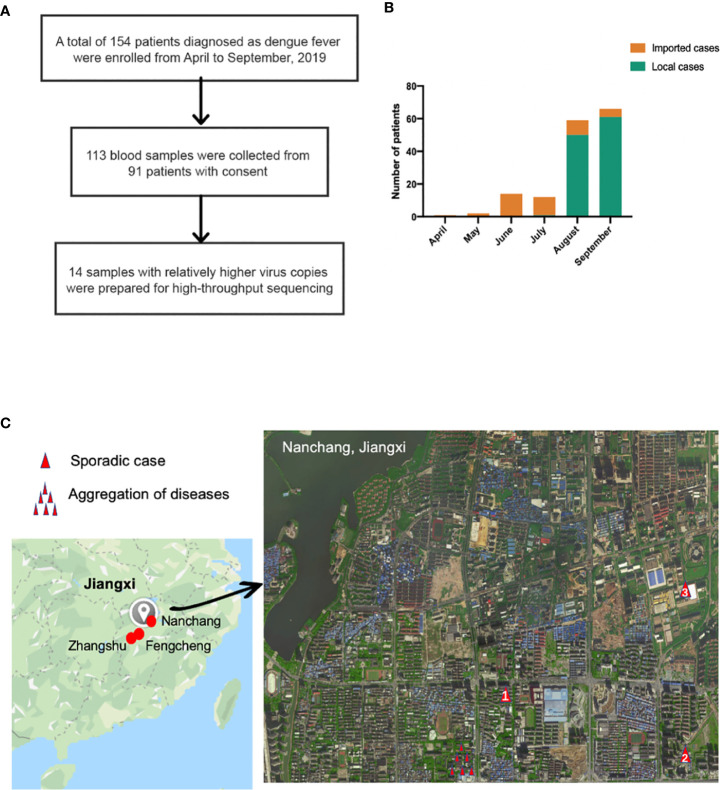
**(A)** Sample enrollment and study flowchart; **(B)** The number of local and imported cases from April to September **(C)** Isolation sites in Jiangxi. Red circles in the left in Jiangxi represented isolation cities: Nanchang, Fengcheng and Zhangshu. For the enlarged Nanchang map, the single red triangle indicated sporadic cases in Nanchang while several triangle symbols meant aggregation of dengue fever. Panel **(C)** was based on maps from https://www.google.com/maps.

A total of 154 patients were enrolled (**Figure 1A**) in the study from April to September 2019, and the baseline characteristics are shown in **Table 1**. Ninety-three, forty-nine, and twelve cases were collected from Nanchang, Zhangshu, and Fengcheng, respectively. Among the 154 cases, forty-two cases were identified as imported cases back from other countries; the majority (39/42) of which came from Cambodia. One hundred and nine cases were identified as local cases, and three cases were domestic cases from other provinces to Jiangxi. We observed a majority of DENV cases in August and September, with 125 cases reported, compared with 1, 2, 14, and 12 cases reported in April, May, June, and July, respectively. As shown in **Figure 1B**, the imported cases appeared in April while local cases emerged in July and increased rapidly in August and September, and small outbreaks were observed in a localized area in Nanchang (**Figure 1C**). The mean onset time (symptoms onset to admission) of enrolled patients was five days (range, 0–15). Approximately 94.16% (145/154) of patients presented with fever, and the proportion of rash, headache, and muscle pains was 31.37%, 20.13%, and 22.08%, respectively. We did not observe mucosal and gastrointestinal bleeding, a disorder of consciousness, shock, or respiratory failure. Overall, the clinical symptoms of all enrolled patients were mild, with no severe symptoms reported. All patients were later relieved during the follow-up monitoring.

**Table 1:** Baseline characteristics of enrolled dengue fever patients.

Characteristics	N
**Sex**	
**Male(n)**	90
**Female(n)**	64
**Age range (years)**	4-84
**Epidemic characteristics**	
Imported cases (n)	42
Cambodia(n)	39
Philippines(n)	1
Thailand(n)	1
Malaysia(n)	1
Domestic input to Jiangxi	3
Local cases	109
Travelling to epidemic area (n, %)	80, 51.95%
History of mosquito bites (n, %)	136, 88.31%
**Onset time (mean, range (d))**	5(0-15)
**Clinical presentations**	
Fever (n)	145
Headache (n)	31
Muscle pains (n)	34
Joint pains (n)	6
Rash (n)	48
Mucosal and gastrointestinal bleeding (n)	0
Disorder of consciousness (n)	0
Shock (n)	0
Respiratory failure (n)	0
**Clinical outcomes**	
Relieved	154, 100%

### Etiological Examination of Dengue Virus During Jiangxi Dengue Outbreak

Overall, 113 samples collected from 91 patients from Jiangxi were analyzed using DENV real-time PCR. A total of 106 samples were identified as DENV-1, two serum samples from one patient were identified as DENV-2, and five samples tested negative. The positive rate of dengue NS1 antigen was 100.00% (37/37), whereas the positive rate of dengue antibody IgM and IgG was 50.00% (11/22) and 13.63% (3/22), respectively.

### Phylogenic and Molecular Analyses of Dengue Virus During Jiangxi Dengue Transmission

**Figure 2:**
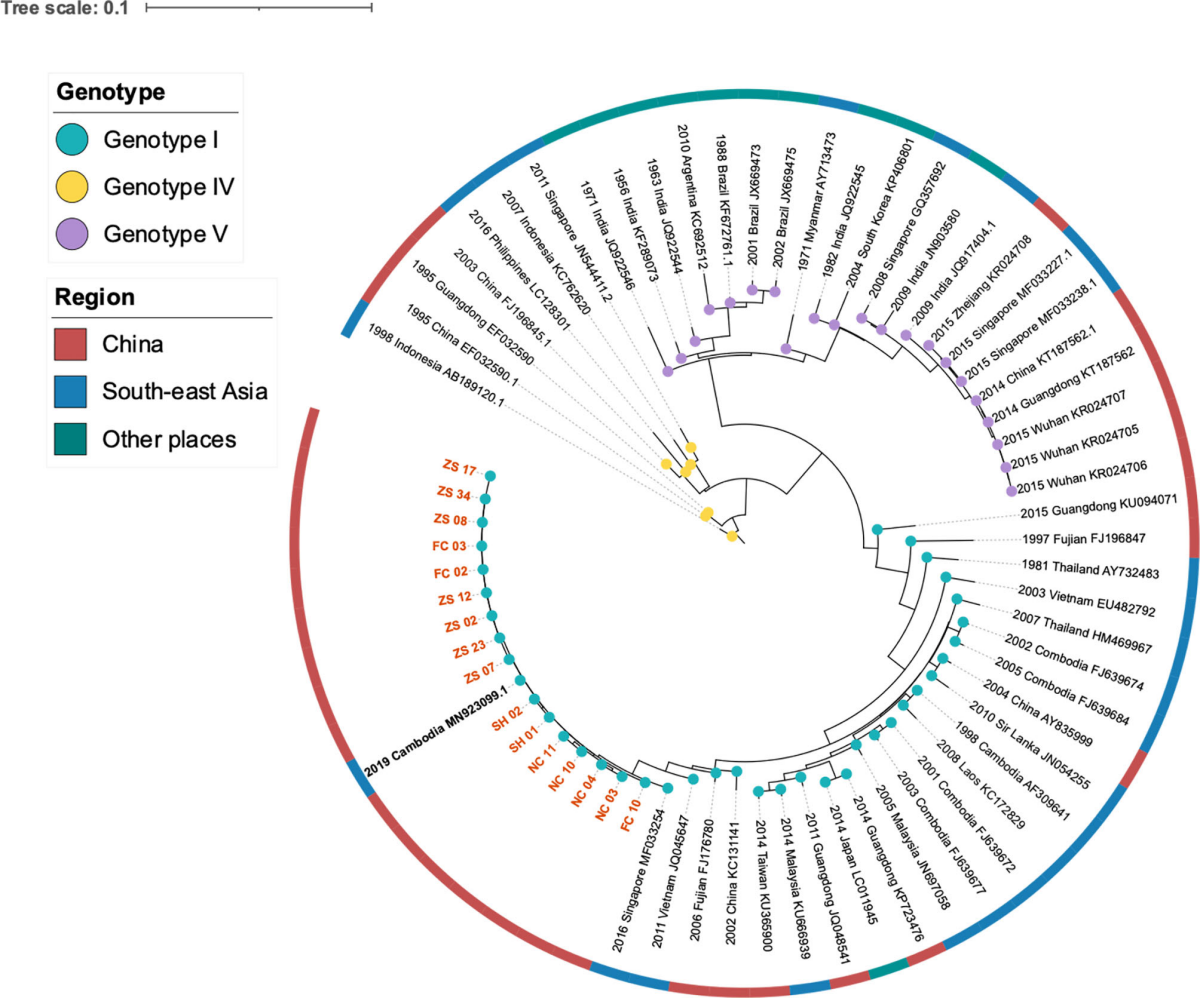
Phylogeny and isolation resources profile of dengue virus type I strains. The colored blue, yellow and purple nodes refereed to DENV Genotype I, IV, V respectively. The dark red, blue and green color strip outside the circular tree indicated isolation resourced of these strains: China, South-east Asia and other places separately. Orange bold branch symbols meant virus we sequenced in this study and the black bold symbol is the strain isolated from a traveler back from Cambodia.

We investigated the nucleotide evolutionary relationships of the dengue virus during the Jiangxi dengue outbreak, with 56 published dengue virus genomes (**Figure 2A**). As illustrated in **Figure 2**, 69 selected sequences were separated into three groups, referring to DENV-1 Genotype I, IV, V, respectively. Sixteen DENV-1 genomes sequenced in this study were classified as DENV-1 Genotype I. Theses sequences owned high similarity to the genome from Cambodia (GenBank MN923099.1). All other Cambodia DENV strains and other southeast Asian DENV strains, including Thailand, Laos, Malaysia DENV isolates, had the same ancestor with the sequenced genomes in our study.

**Figure 3:**
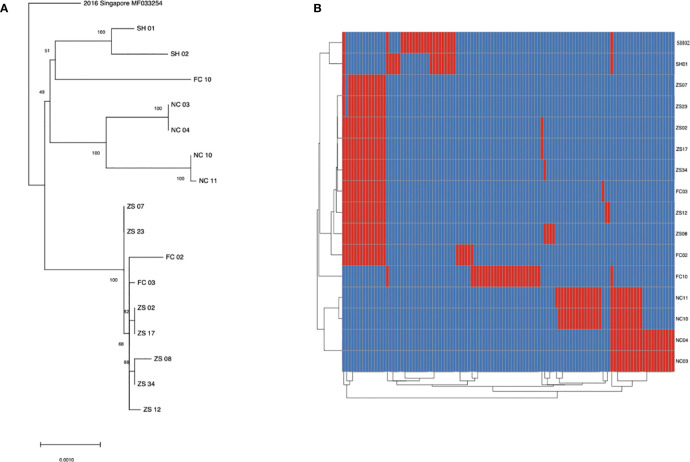
SNP analysis among 16 sequenced dengue virus strains. **(A)** phylogenic tree constructed using 16 virus strains isolated from Jiangxi and Shanghai. The ZS, NC and FC referred to isolates from Zhangshu, Nanchang and Fengcheng separately. The SH 01 and 02 included two isolates from Shanghai. **(B)** A total of 114 SNPs was shown among 16 sequenced genomes were shown. Blue stripes indicated referenced single nucleotide polymorphisms and red ones indicated substitutions in sequenced genomes.

To learn the origin and high-resolution molecular characteristics of this dengue outbreak, we conducted phylogenetic analyses of 16 genomes. A total of 114 SNPs among 16 genomes were detected, indicating the existence of epidemic transmission and microevolution in Jiangxi. **Figure 2B** showed that Zhangshu virus strains, FC02 and FC03, were located in the upper branch. DENV genome NC 03, 04, 10, and 11 from Nanchang were classified into the lower branch. Three pairs of DENV genomes (NC03, NC04; ZS02, ZS17, and ZS07, ZS23) were found to be identical (**Figure 3**). The sequence-based findings were consistent with an epidemiological investigation into this transmission. NC03 and NC04 were from patients living in the same village in Nanchang, Jiangxi while ZS02, ZS17, and ZS07, ZS23 were from patients living in villages that were located within two kilometers.

To reveal evolutionary differences among amino acid sequences and functional changes, we next performed a phylogenetic analysis of 69 amino acid sequences, and showed that the evolutionary relationships are consistent with that of nucleotide genome results (**Supplementary Figure 2**). Sixty-nine amino acid sequences were classified into three categories: DENV-1 Genotype I, IV, V from upper to lower branches. Fourteen sequences collected from Jiangxi and two from Shanghai were closely related to the sequence of a traveler back from Cambodia in 2019 (GenBank MN923099.1). Analysis of detailed alterations of amino acids revealed that compared with the reference amino acid sequence from Singapore in 2016, 20 synonymous amino acid alterations were found in 16 DENV-1 sequences involved in this outbreak (**Supplementary Table 4**). Three out of 20 codon alterations in all 16 sequences were annotated with functional changes (**Table 2**).

**Table 2:** Three common non-synonymous amino acid mutations in 16 sequenced samples comparing to reference sequence NP059433.

Reference Amino acid sequence	Position	Protein	Reference codon	Altered codon	Reference amino acid	Alteredamino acid
NP059433	2437	NS1	G	A	AUG (Methionine)	AUA (Isoleucine)
NP059433	2750	NS1	T	C	UAC (Tyrosine)	CAC (Histidine)
NP059433	3902	NS2A	C	T	CAU (Histidine)	TAU (Tyrosine)

The three altered positions were located in NP059433 position 2437 (AUG→AUA, Methionine →Isoleucine), 2750 (UAC→CAC, Tyrosine→ Histidine), and 3902 (CAU→TAU, Histidine→ Tyrosine). Altered positions 2437 and 2750 were located in NS1 protein, whereas 3902 was located in NS2A protein.

### Mosquito’s Density and Climate Characteristics of Jiangxi

**Figure 4:**
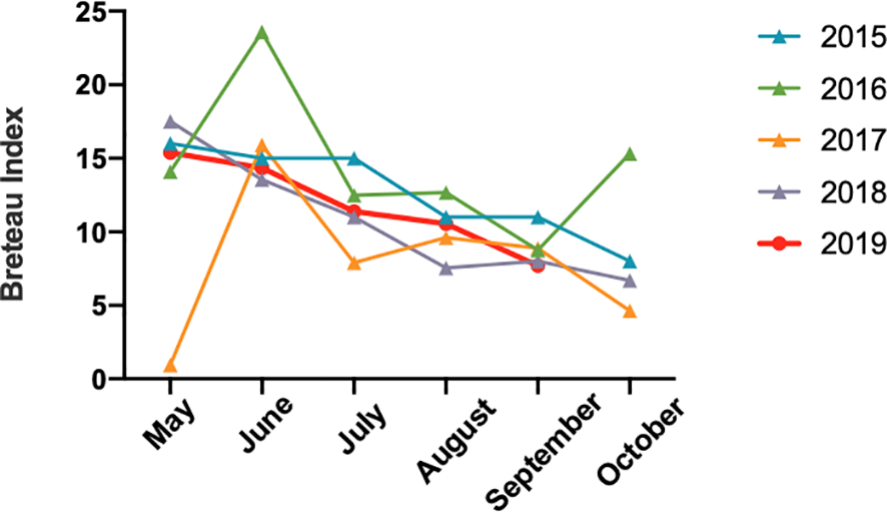
The mosquito Breteau Index of Jiangxi from May to October between 2015 and 2019.

Based on epidemiological investigations, the average monthly temperature and rain value indicated that Jiangxi had experienced water shortages in 2019, and the July to October temperatures were the highest since 1961 (http://www.ecns.cn/news/2019-11-08/detail-ifzqrxfh5725155.shtml). Surprisingly, Breteau Index, which represents mosquito density (Aryaprema and Xue, 2019) (**Figure 4**) showed no differences from previous years.

## Discussion

This work analyzed the clinical and epidemiology characteristics of 154 dengue fever cases in Jiangxi, a low-epidemic region in south-east China and molecular data of virus strains. The data present clear findings: (i) dengue virus strains shared close phylogenetic relationships (ii) the emergence of dengue cases led to autochthonous transmission in several cities in Jiangxi.

Although phylogenic and evolutionary analyses illustrated a high similarity of the dengue virus strains, a total of 97 synonymous mutations and 17 non-synonymous mutations were detected among 16 strains, suggesting fast variations of dengue virus during transmission. Moreover, comparison to referenced NP059433 revealed three common alterations of amino acids, further suggesting the evolution of dengue virus. The locations of the three altered amino acids were correlated with early viral RNA replication and innate immune system activation (Guzman et al., 2016).

We evaluated the mosquito density to investigate the conversion of Jiangxi into a dengue fever epidemic region, but did not show obvious change compared with those in previous years. For climate change, we found that the summer in 2019 experienced a higher temperature and water shortages. According to previous reports, high temperatures and humid climate could increase the incidence and transmission of dengue fever (Ebi and Nealon, 2016). Thus, the higher temperature in 2019 might accelerate the dengue outbreak.

From clinical data, the cases from April to June were all imported cases and most of them were back from Cambodia. Local cases emerged in July and increased rapidly in August and September. What’s more, a new airline connecting Preah Sihanouk, Cambodia, and Nanchang, Jiangxi, has been operational since June 1, 2019. Combined with epidemiologic information of travelers in Jiangxi last year, we deduced that the emergence of the Jiangxi dengue outbreak might be linked to the Cambodia dengue wave. Further, the phylogenetic tree showed a high similarity of Jiangxi strains and Cambodia strain, and this evidence provided further evidence of our conclusion based on the clinical and epidemiological evaluation. We speculate that the new airline between Nanchang and Cambodia may be the contributory factor of a continuous movement of dengue virus-infected individuals traveling from Cambodia, therefore causing a sharp rise in the DENV infection cases in Jiangxi since June 2019. Air travel was reported to have a measurable impact on the importation of dengue virus to China, and prediction of dengue virus importation could help prevent and control the spread of the disease (Findlater et al., 2019; Wilson, 2019).

The aggregation of diseases in a small village in Nanchang, after a few sporadic cases in the nearby areas, revealed disease transmission pattern. It emphasized on the importance of regional prevention and control of dengue fever in this era of rapid growth of worldwide traffic. Additionally, areas previously considered non-epidemic should not be neglected.

As dengue fever have an incubation period from 3 to 14 days (Guzman et al., 2016), it is challenging for healthcare staff to control the disease in time, especially imported cases. Any place, especially humid tropical and subtropical areas that receive travelers and visitors from dengue fever epidemic areas should institute stringent prevention and control strategies. The silent majority carriers in the incubation period were found to be major sources of dengue virus transmission (Ferguson et al., 2018). What’s more, live attenuated vaccine (CYD-TDV) has been used and candidate dengue vaccinations are currently proven to be effective in endemic populations (Biswal et al., 2019; Biswal et al., 2020; Deng et al., 2020; Tricou et al., 2020).

It has seemed that, during the human fight against the mosquito-borne virus, our deep understanding of diseases has failed to provide us with the satisfying tool to eliminate or, at least, prevent outbreaks of the disease. Therefore, this phenomenon suggests the great necessity of continuously studying the disease’s epidemic traits and the possible underlying reasons.

This study has several limitations. First, we collected serum samples from July to September since the emergence of local transmission; thus, samples before July could not be included in the analysis. Second, metagenomic next-generation sequencing was performed successfully in genomes with relatively higher viral copies, whereas other samples failed to produce enough reads for analysis because of low viral loads. The limited sample size and sequenced data hinder precise and clear determination of the transmission route.

## Conclusions

In conclusion, this study demonstrated that the emergence of dengue cases led to autochthonous transmission in several cities in Jiangxi. With increasing globalization, there is a need for more stringent disease prevention and control measures in regions with visitors and travelers from dengue fever epidemic areas.

## Data Availability Statement

The datasets presented in this study can be found in online repositories. The names of the repository/repositories and accession number(s) can be found in the article/**Supplementary Material**.

## Author Contributions

This study was designed and supervised by JA, WZ and NJ. YZha, JA and NJ wrote the paper. YZha and JWu conducted RT-PCR, antigen and antibody tests. YZho carried out metagenomic library construction. HC, SW, XW, XT, YH, WJ, XP, JR, LW, JWa, QC and FL collected and analyzed clinical and epidemiologic data. All authors contributed to the article and approved the submitted version.

## Funding

This study was supported by National Natural Science Foundation of China [grant number 82041010], Key Technologies Research and Development Program for Infectious Diseases of China [2018ZX10305-409-001-003] and Shanghai Youth Science and Technology Talents Sailing Project [20YF1404300].

## Conflict of Interest

The authors declare that the research was conducted in the absence of any commercial or financial relationships that could be construed as a potential conflict of interest.
