# Supplementary material for: Emergence and Autochthonous Transmission of Dengue Virus Type I in a Low-Epidemic Region in Southeast China
Source: Front Cell Infect Microbiol. 2021 Mar 24;11:638785. doi: 10.3389/fcimb.2021.638785 (PMC8024628; doi:10.3389/fcimb.2021.638785)
Supplement: Supplementary file 1 [file DataSheet_1.docx]

**Supplementary file**

**Suppl. Table 1. The number of dengue fever reported dengue fever worldwide.**

**Suppl. Table 2. Sample list of NS1 antigen and antibody test.**

**Suppl. Table 3. DENV result of Dengue virus using real-time PCR.**

**Suppl. Table 4. Synonymous amino acid alterations detected in 16 DENV-1 sequences involved in this outbreak comparing to NP059433.** The orange stripe meant 3 out of 20 codon alterations could be found in all 16 sequences with functional changes

**Suppl. Figure 1. National reported cases of Dengue fever from 2016-2019 in China.**

**Suppl. Figure 2. Correlation heatmap of 69 dengue amino acid sequences.** The four colors referred to nonpolar amino acid and three types of polar amino acid.

**Suppl. Table 1. The number of dengue fever reported dengue fever worldwide.**

| **To date** | **Country** | **Number of Cases** | **Number of deaths** |
| --- | --- | --- | --- |
| 2019/08/24 | Philippines [1] | 249,332 | 1021 |
| 2019/07/27 | Vietnam [2] | 124,751 | 15 |
| 2019/08/17 | Malaysia [3] | 85,270 | 121 |
| 2019/09/24 | Bangladesh [4] | 85,757 | 75 |
| 2019/09/09 | Thailand [5] | 4,903 | 3 |
| 2019/09/13 | Sri Lanka [6] | 46,896 | - |
| 2019/08/17 | Laos [3] | 24,758 | 51 |
| 2019/07/14 | Cambodia [3] | 21,130 | 25 |
| 2019/02/04 | Indonesia [7] | 16,692 | 176 |
| 2019/08/10 | Myanmar [8] | 13,048 | 68 |
| 2019/09/18 | Singapore [3] | 12,000 | - |
| 2019/04/30 | Maldives [9] | 1,957 | - |
| 2019/08/05 | America [10] | >2,000,000 | 723 |

**Suppl. Table 2. Sample list of NS1 antigen and antibody test.**

| *Sample ID* | *NS1 antigen* | *Antibody* |
| --- | --- | --- |
| *NC 09* | *√* | *√* |
| *NC 10* | *√* | *√* |
| *NC 11* | *√* | *√* |
| *NC 15* | *√* | *√* |
| *NC 16* | *√* | *√* |
| *NC 17* | *√* | *√* |
| *NC 19* | *√* | *√* |
| *NC 20* | *√* | *√* |
| *NC 21* | *√* | *√* |
| *NC 22* | *√* | *√* |
| *NC 23* | *√* | *√* |
| *NC 24* | *√* | *√* |
| *NC 25* | *√* | *√* |
| *NC 26* | *√* | *√* |
| *NC 27* | *√* | *√* |
| *NC 28* | *√* | *√* |
| *NC 29* | *√* |  |
| *NC 30* | *√* | *√* |
| *NC 31* | *√* | *√* |
| *NC 32* | *√* | *√* |
| *NC 33* | *√* | *√* |
| *NC 34* | *√* | *√* |
| *NC 35* | *√* | *√* |
| *FC 01* | *√* |  |
| *FC 02* | *√* |  |
| *FC 03* | *√* |  |
| *FC 04* | *√* |  |
| *FC 05* | *√* |  |
| *FC 06* | *√* |  |
| *FC 07* | *√* |  |
| *FC 08* | *√* |  |
| *FC 09* | *√* |  |
| *FC 10* | *√* |  |
| *FC 11* | *√* |  |
| *FC 12* | *√* |  |
| *ZS 65* | *√* |  |
| *ZS 70* | *√* |  |

**Suppl. Table 3. DENV result of Dengue virus using real-time PCR.**

| **Sample ID** | **Isolation City** | **DENV  result** |
| --- | --- | --- |
| FC01 | Fengcheng | DENV-1 |
| FC02 | Fengcheng | DENV-1 |
| FC03 | Fengcheng | DENV-1 |
| FC04 | Fengcheng | DENV-1 |
| FC05 | Fengcheng | DENV-1 |
| FC06 | Fengcheng | DENV-1 |
| FC07 | Fengcheng | DENV-1 |
| FC08 | Fengcheng | DENV-1 |
| FC09 | Fengcheng | DENV-1 |
| FC10 | Fengcheng | DENV-1 |
| FC11 | Fengcheng | DENV-1 |
| FC12 | Fengcheng | DENV-1 |
| NC 31 | Nanchang | DENV-1 |
| NC 32 | Nanchang | DENV-1 |
| NC 33 | Nanchang | DENV-1 |
| NC 34 | Nanchang | DENV-1 |
| NC 35 | Nanchang | Negative |
| NC01 | Nanchang | DENV-1 |
| NC02 | Nanchang | DENV-1 |
| NC03 | Nanchang | DENV-1 |
| NC04 | Nanchang | DENV-1 |
| NC05 | Nanchang | DENV-1 |
| NC06 | Nanchang | DENV-1 |
| NC07 | Nanchang | DENV-1 |
| NC08 | Nanchang | DENV-1 |
| NC09 | Nanchang | DENV-1 |
| NC10 | Nanchang | DENV-1 |
| NC11 | Nanchang | DENV-1 |
| NC12 | Nanchang | DENV-1 |
| NC13 | Nanchang | DENV-1 |
| NC14 | Nanchang | DENV-1 |
| NC15 | Nanchang | DENV-1 |
| NC16 | Nanchang | DENV-1 |
| NC17 | Nanchang | DENV-1 |
| NC18 | Nanchang | Negative |
| NC19 | Nanchang | DENV-1 |
| NC20 | Nanchang | DENV-1 |
| NC21 | Nanchang | DENV-1 |
| NC22 | Nanchang | DENV-1 |
| NC23 | Nanchang | DENV-1 |
| NC24 | Nanchang | DENV-1 |
| NC25 | Nanchang | Negative |
| NC26 | Nanchang | DENV-1 |
| NC27 | Nanchang | DENV-1 |
| NC28 | Nanchang | DENV-1 |
| NC29 | Nanchang | DENV-1 |
| NC30 | Nanchang | DENV-1 |
| YC02 | Zhangshu | DENV-1 |
| YC03 | Zhangshu | DENV-1 |
| YC04 | Zhangshu | DENV-1 |
| YC05 | Zhangshu | DENV-1 |
| YC06 | Zhangshu | DENV-1 |
| YC07 | Zhangshu | DENV-1 |
| YC08 | Zhangshu | DENV-1 |
| YC09 | Zhangshu | DENV-1 |
| YC10 | Zhangshu | DENV-1 |
| YC11 | Zhangshu | DENV-1 |
| YC12 | Zhangshu | DENV-1 |
| YC13 | Zhangshu | DENV-1 |
| YC14 | Zhangshu | DENV-1 |
| YC15 | Zhangshu | DENV-1 |
| YC16 | Zhangshu | DENV-1 |
| YC17 | Zhangshu | DENV-1 |
| YC18 | Zhangshu | DENV-1 |
| YC19 | Zhangshu | DENV-1 |
| YC20 | Zhangshu | DENV-1 |
| YC21 | Zhangshu | DENV-1 |
| YC23 | Zhangshu | DENV-1 |
| YC24 | Zhangshu | DENV-1 |
| YC25 | Zhangshu | DENV-1 |
| YC26 | Zhangshu | DENV-1 |
| YC27 | Zhangshu | DENV-1 |
| YC28 | Zhangshu | DENV-1 |
| YC29 | Zhangshu | DENV-1 |
| YC30 | Zhangshu | DENV-1 |
| YC31 | Zhangshu | DENV-1 |
| YC33 | Zhangshu | DENV-1 |
| YC34 | Zhangshu | DENV-1 |
| YC35 | Zhangshu | DENV-1 |
| YC36 | Zhangshu | DENV-1 |
| YC37 | Zhangshu | DENV-1 |
| YC38 | Zhangshu | DENV-1 |
| YC39 | Zhangshu | DENV-1 |
| YC40 | Zhangshu | DENV-1 |
| YC41 | Zhangshu | Negative |
| YC42 | Zhangshu | DENV-1 |
| YC43 | Zhangshu | DENV-1 |
| YC44 | Zhangshu | DENV-1 |
| YC45 | Zhangshu | DENV-1 |
| YC47 | Zhangshu | DENV-1 |
| YC48 | Zhangshu | DENV-1 |
| YC51 | Zhangshu | DENV-1 |
| YC52 | Zhangshu | DENV-1 |
| YC53 | Zhangshu | DENV-1 |
| YC54 | Zhangshu | DENV-1 |
| YC55 | Zhangshu | DENV-1 |
| YC56 | Zhangshu | DENV-1 |
| YC58 | Zhangshu | DENV-1 |
| YC59 | Zhangshu | DENV-1 |
| YC60 | Zhangshu | Negative |
| YC61 | Zhangshu | DENV-1 |
| YC62 | Zhangshu | DENV-1 |
| YC63 | Zhangshu | DENV-1 |
| YC64 | Zhangshu | DENV-1 |
| YC65 | Zhangshu | DENV-1 |
| YC66 | Zhangshu | DENV-2 |
| YC67 | Zhangshu | DENV-1 |
| YC68 | Zhangshu | DENV-1 |
| YC69 | Zhangshu | DENV-2 |
| YC70 | Zhangshu | DENV-1 |
| YC71 | Zhangshu | DENV-1 |
| YC72 | Zhangshu | DENV-1 |
| YC73 | Zhangshu | DENV-1 |

**Suppl. Table 4. Synonymous amino acid alterations detected in 16 DENV-1 sequences involved in this outbreak comparing to NP059433.** The orange stripe meant 3 out of 20 codon alterations could be found in all 16 sequences with functional changes comparing to NP059433.

| CHR | POS | REF | ALT | Reference | ALT1 | Reference amino acid | Alteration amino acid |
| --- | --- | --- | --- | --- | --- | --- | --- |
| MF033254.1 | 410 | C | A | CUC | AUC | L | I |
| MF033254.1 | 689 | G | A | GAG | AAG | E | K |
| MF033254.1 | 1301 | A | T | ACA | TCA | T | S |
| MF033254.1 | 1379 | C | T | CAC | TAC | H | Y |
| MF033254.1 | 1947 | C | T | UCG | UTG | S | L |
| MF033254.1 | 2019 | A | G | GAA | GGA | E | G |
| MF033254.1 | 2090 | G | A | GCU | ACU | A | T |
| MF033254.1 | 2406 | T | C | GUC | GCC | V | A |
| **MF033254.1** | **2437** | **G** | **A** | **AUG** | **AUA** | **M** | **I** |
| **MF033254.1** | **2750** | **T** | **C** | **UAC** | **CAC** | **Y** | **H** |
| **MF033254.1** | **3902** | **C** | **T** | **CAU** | **TAU** | **H** | **Y** |
| MF033254.1 | 4776 | A | G | CAA | CGA | Q | R |
| MF033254.1 | 5021 | G | A | GCU | ACU | A | T |
| MF033254.1 | 5564 | T | C | UAU | CAU | Y | H |
| MF033254.1 | 6398 | T | A | UUA | AUA | L | I |
| MF033254.1 | 6509 | A | G | AUG | GUG | M | V |
| MF033254.1 | 7283 | G | A | GCA | ACA | A | T |
| MF033254.1 | 9480 | C | T | CCA | CTA | P | L |
| MF033254.1 | 9512 | G | A | GAA | AAA | E | K |
| MF033254.1 | 10065 | G | A | AGU | AAU | S | N |

**Suppl. Figure 1. National reported cases of Dengue virus infection from 2016-2019 in China.**


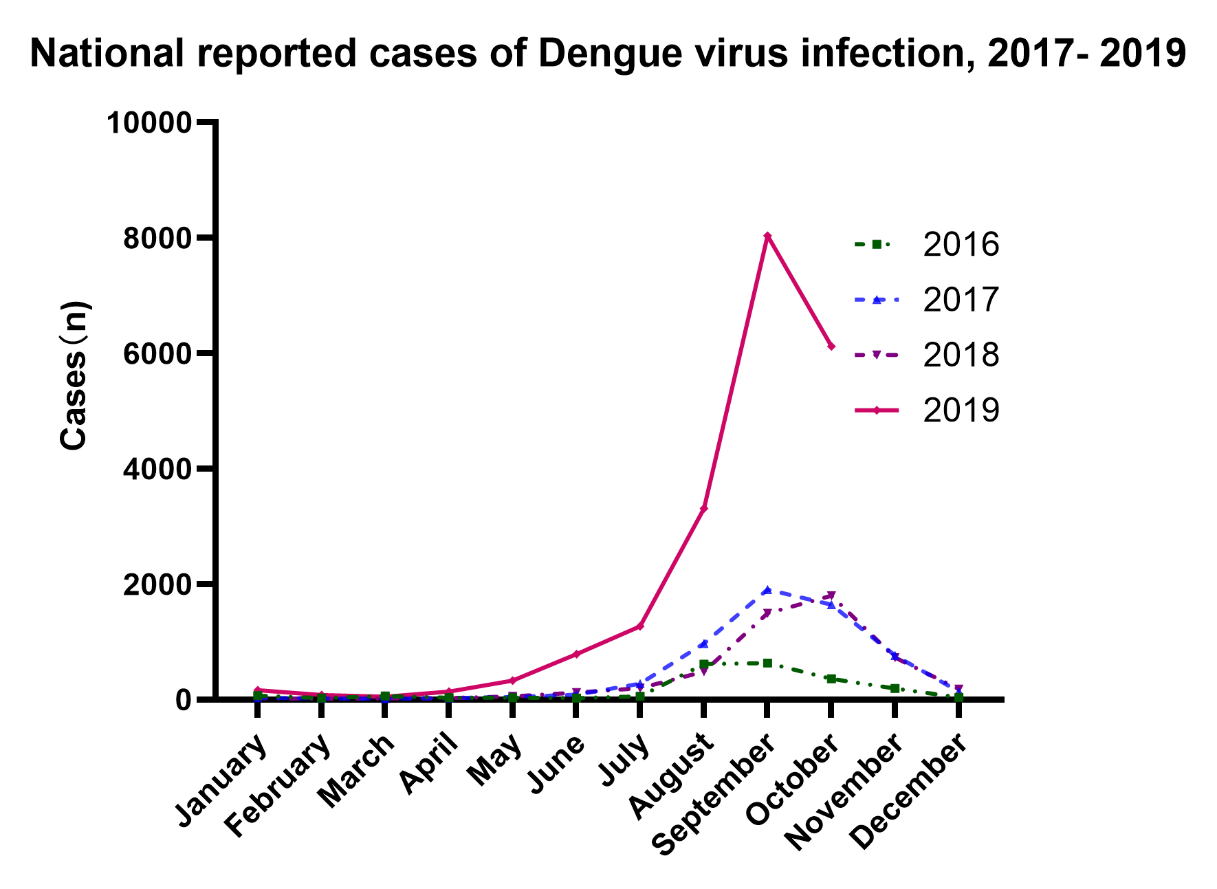


**Suppl. Figure 2. Correlation heatmap of 69 dengue amino acid sequences.** The four colors referred to nonpolar amino acid and three types of polar amino acid.

**Reference**

[1] http://www.china.org.cn/world/2019-09/11/content_75194850.htm

[2] <https://outbreaknewstoday.com/vietnam-dengue-update-dengue-forecasting-system-32895/>

[3]https://www.who.int/docs/default-source/wpro---documents/emergency/surveillance/dengue/dengue-20200507.pdf?sfvrsn=5160e027_26

[4] <https://reliefweb.int/sites/reliefweb.int/files/resources/03-08-19-Dengue%20Health%20Bulletin_Volume%202_CDC.pdf>

[5] <https://thailandbloggers.com/2019/09/09/dengue-fever-in-thailand-3-deaths-from-january-2019-in-bangkok/>

[6] http://www.dengue.health.gov.lk

[7] https://www.thejakartapost.com/news/2019/02/06/dengue-death-toll-rises-to-176.html

[8] https://reliefweb.int/report/myanmar/dengue-fever-kills-68-myanmar-8-months

[9] <https://www.sun.com.mv/53641>

[10] https://medicalxpress.com/news/2019-08-dengue-fever-outbreak-latin-america.html
